# High-numerical-aperture macroscope optics for time-resolved experiments

**DOI:** 10.1107/S1600577519004119

**Published:** 2019-05-21

**Authors:** Minna Bührer, Marco Stampanoni, Xavier Rochet, Felix Büchi, Jens Eller, Federica Marone

**Affiliations:** a Swiss Light Source, Paul Scherrer Institut, Forschungsstrasse 111, Villigen 5232, Switzerland; b Institute for Biomedical Engineering, University and ETH Zürich, Zürich 8092, Switzerland; c Optique Peter, Lentilly 69210, France; d Electrochemistry Laboratory, Paul Scherrer Institut, Forschungsstrasse 111, Villigen 5232, Switzerland

**Keywords:** X-ray tomographic microscopy, high numerical aperture optics, optics characterization, image quality

## Abstract

A novel high-quality custom-made macroscope optics, dedicated to high-resolution time-resolved X-ray tomographic microscopy at the TOMCAT beamline at the Swiss Light Source (Paul Scherrer Institut, Switzerland), is introduced. The macroscope offers 4× magnification, has a very high numerical aperture of 0.35 and it is modular and highly flexible. It can be mounted both in a horizontal and vertical configuration, enabling imaging of tall samples close to the scintillator, to avoid edge-enhancement artefacts. The macroscope performance was characterized and compared with two existing in-house imaging setups, one dedicated to high spatial and one to high temporal resolution. The novel macroscope shows superior performance for both imaging settings compared with the previous systems. For the time-resolved setup, the macroscope is 4 times more efficient than the previous system and, at the same time, the spatial resolution is also increased by a factor of 6. For the high-spatial-resolution setup, the macroscope is up to 8.5 times more efficient with a moderate spatial resolution improvement (factor of 1.5). This high efficiency, increased spatial resolution and very high image quality offered by the novel macroscope optics will make 10–20 Hz high-resolution tomographic studies routinely possible, unlocking unprecedented possibilities for the tomographic investigations of dynamic processes and radiation-sensitive samples.

## Abstract 2

A novel high quality, high numerical aperture custom-made macroscope optics makes 10–20 Hz high-resolution tomographic studies at TOMCAT routine. The macroscope performance was characterized and found to be superior compared with previous high-spatial- and high-temporal-resolution setups available in-house: the new macroscope is 4 to 8 times more efficient and the spatial resolution is improved by up to a factor of 6.

## 1. Introduction   

X-ray tomographic microscopy offered at third-generation synchrotron sources enables investigations of a variety of samples in a fast and high-resolution manner. Technological developments (*e.g.* in CMOS detection systems) during the past decade have enabled extending this technique from the study of purely static specimens to dynamic processes, offering unprecedented three-dimensional insight into phenomena such as the internal mechanics of a blowfly (Walker *et al.*, 2014 ▸), bubble growth in basaltic foams (Baker *et al.*, 2012 ▸) and material fracture processes during *in situ* tensile tests (Maire *et al.*, 2016 ▸).

The beamline for TOmographic Microscopy and Coherent rAdiology experimenTs (TOMCAT) at the Swiss Light Source at the Paul Scherrer Institut, Switzerland (Stampanoni *et al.*, 2006 ▸), with its endstation dedicated to time-resolved experiments (Mokso *et al.*, 2010 ▸), has been at the forefront in these developments. The acquisition time of a single tomogram has decreased from several minutes (*e.g.* Lambert *et al.*, 2007 ▸) to fractions of a second (*e.g.* Mokso *et al.*, 2013 ▸; dos Santos Rolo *et al.*, 2014 ▸; Maire *et al.*, 2016 ▸; Ruhlandt *et al.*, 2017 ▸). A new in-house-developed read-out system has made sustained tomographic experiments a reality with a continuous data stream of nearly 8 GB s^−1^ (Mokso *et al.*, 2017 ▸), significantly outperforming commercial systems. Although dynamic studies with sub-second acquisition time per tomogram are routinely performed at TOMCAT (*e.g.* Eller *et al.*, 2015 ▸; Maire *et al.*, 2016 ▸; Ruhlandt *et al.*, 2017 ▸), the achieved spatial resolution is still limited to tens of micrometres for the fastest experiments or, in other words, higher resolution in the micrometre range is possible only for slower experiments.

Since the available flux at the beamline will not increase until the foreseen upgrade of the Swiss Light Source (Streun *et al.*, 2018 ▸) for 2023–2024, a new highly efficient optical component is key for pushing dynamic tomographic studies to the next level. High efficiency is essential for time-resolved studies, particularly when polychromatic radiation is used, as samples and processes under investigation are often sensitive to radiation damage.

Professional photographic lenses for imaging under low light conditions feature a high numerical aperture (up to 0.4), but the image quality they provide does not match the requirements of high-resolution X-ray microscopy (see below). Commercial microscope objectives perform better in terms of image quality, but for moderate magnifications (4–5×) their numerical aperture is limited to 0.1–0.17. Recently, a custom-made high-numerical-aperture 5× magnification objective, mainly dedicated to neutron imaging, has been presented (Trtik & Lehmann, 2016 ▸). In its characterization, focus has though concentrated on the beneficial effects of new scintillator developments, crucial when neutrons are used, rather than on the new optics itself.

Here, we propose a new very high quality and flexible custom-made 4× macroscope, with a high numerical aperture of 0.35, compatible with large chip sensors. These unique features coupled to the recent developments in detector systems (Mokso *et al.*, 2017 ▸) will unlock unprecedented opportunities in high-resolution, time-resolved investigations.

In Section 2, we present the novel macroscope and its technical details, including the optical design and flexible configuration possibilities. In Section 3, we characterize the macroscope performance for different imaging setups and in Section 4 we compare it with two existing imaging setups at TOMCAT, one dedicated to high temporal and one to high-spatial-resolution imaging.

## 2. Macroscope description   

To extend the time-resolved activities at the TOMCAT beamline towards true spatial resolutions of a few micrometres, while still having a field of view sufficiently large for capturing representatively large volumes of dynamic samples and processes, a 4× magnification has been specified for this new optics, compatible with sensor chips with a diagonal of more than 30 mm. The highest efficiency possible has been striven for; it turned out that the maximum numerical aperture still guaranteeing the highest image quality (*e.g.* no vignetting) was 0.35. Time-resolved studies often require special sample environments, such as chambers; so to be able to accommodate the widest variety of sample geometries without having to compromise the image quality we envisaged a modular macroscope design. In the following, we present the optical design, the manufacturing process, the optical tests, the motorized aperture and the flexible configuration possibilities in more detail.

### 2.1. Optical design   

#### 2.1.1. Specifications   

To achieve the highest quality, the optical formula for this very high (0.35) numerical aperture optics had to provide a modulation transfer function close to the diffraction limit and ensure no geometrical distortions. Real glass characteristics, environment temperature in the beamline hutch and realistic manufacturing and assembling tolerances have been considered in the optical design. To allow the use of scientific cameras with large sensors, an image diameter of 44 mm free of corner vignetting has been specified. The system should be apochromatic to be able to use scintillator screens emitting at different wavelength in the visible bandwidth.

For compatibility with the high flux of polychromatic radiation at a third-generation synchrotron source, a long working distance is required to bend the optical path between the scintillator and the objective. Sufficient space for mounting a radiation-resistant X-ray protective window in front of the objective is also needed. The objective should though remain compact enough to be able to analyse tall specimens with a diameter up to 50 mm close to the scintillator screen. A motorized aperture diaphragm at the rear of the objective is also foreseen to increase the objective depth of focus when thick scintillators are used. This diaphragm is not used to improve the image quality, because the objective is designed to achieve maximum resolution at full aperture.

#### 2.1.2. Realization   

To keep maximum flexibility and enable future upgrade to a different magnification, a tandem design combining a front infinity corrected objective coupled with a tube lens has been chosen. This configuration also allows for easier focusing by translation of the front objective inside the housing. It also leads to a more compact macroscope front part, facilitating work with larger specimens and *in situ* cells.

**Figure 1:**
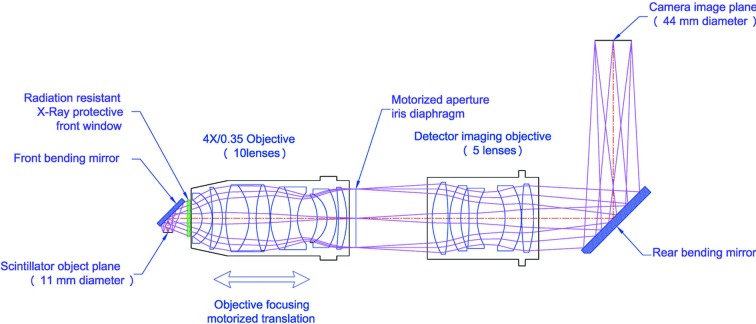
The macroscope structure illustrated in the horizontal setup.

**Figure 2:**
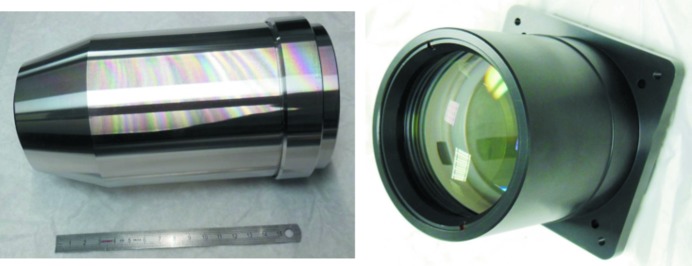
Macroscope optical components: front infinity corrected objective (left) and tube lens (right).

**Figure 3:**
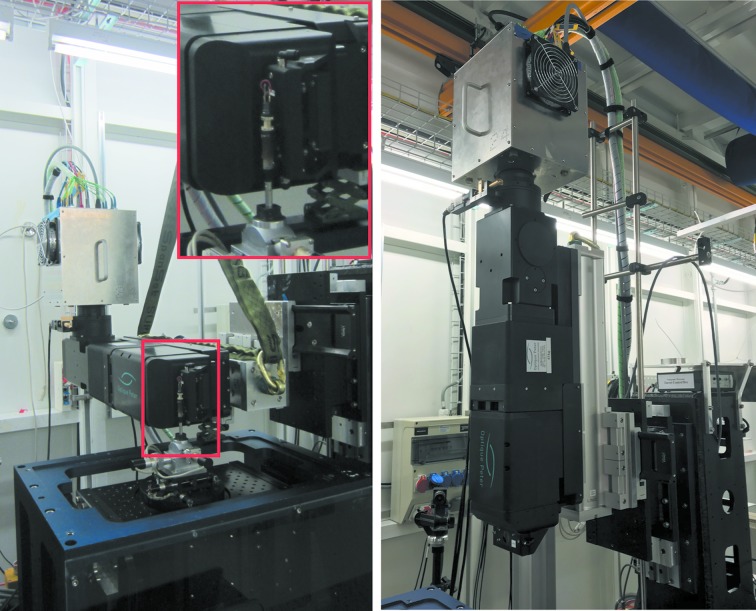
The novel macroscope setup. Left: the macroscope coupled with the GigaFRoST camera in horizontal configuration. Right: the macroscope coupled with the GigaFRoST camera in vertical configuration.

The macroscope (Fig. 1 ▸) consists of a scintillator support from where the light is directed by a front bending mirror through the radiation-resistant X-ray protective front window to the objective. The objective (Fig. 2 ▸) consists of ten lenses, each specifically designed and polished to achieve the high numerical aperture of 0.35 and high-quality images. The focal length of this objective is 100 mm. A motorized diaphragm is located at the exit pupil after the objective to adjust its depth of focus depending on the thickness of the chosen scintillator. The diaphragm is followed by a filter support that can accommodate up to 8 mm-thick filters. When the focus is adjusted, the objective is moved together with the diaphragm and the filter with respect to the scintillator. A detector imaging objective (Fig. 2 ▸), also known as a tube lens, is located after the aperture and consists of five lenses. Its focal length is 400 mm, resulting in a fixed 4× magnification of the combined optics. The macroscope has been designed to work in two different geometrical configurations (Fig. 3 ▸). In standard vertical configuration, the tube lens is directly followed by the camera imaging plane; in standard horizontal setup, an additional rear bending mirror is needed to redirect light towards a camera imaging plane located on the side of the macroscope, facing upwards in the horizontal configuration.

### 2.2. Manufacturing details   

Very high quality exotic glasses have been chosen to ensure the requested lens performance. Some glasses had to be manufactured upon a specific order and glass pallets were melted to the specific lens dimensions. To reach performances very close to the theoretical optical formula, the optical formula originally computed with catalogue glass characteristics has been adapted to correct for variations in the measured optical characteristics for each glass production batch.

Each lens has been polished manually to reach very high precision (λ/10 typical, which in our case is 53.5 nm). The geometry of each lens has been measured and the optical formula adapted, by adjusting the mechanical distance between some lenses, to account for manufacturing imperfections. Due to the very high curvature of some lenses and the fragility of the glass, few lenses broke during polishing.

Doublets and triplets were assembled on a high-precision rotation stage with micrometre precision. The mounting facility included also metrology tools and laser optical alignment systems to measure the assembly precision. The objectives were assembled on the same high-precision rotation stage. Each lens was mounted on a mechanical support ring and glued to the previous ring. Due to the drying time of the glue, it was possible to only assemble one lens per day. If the lens had moved during drying, the element had to be dis­assembled, cleaned and the operation redone.

### 2.3. Optical tests   

Prior to delivery, the macroscope performance was tested at the Optique Peter premises using visible light. For this purpose, a resolution test sample (1951 USAF Hi-Resolution Target) was positioned directly in front of the objective. In a first step, the macroscope was coupled to a camera with a small pixel size (1.67 µm; UI-1492LE camera, IDS Imaging Development Systems GmbH, Obersulm, Germany) to test the resolution limits of the new optics. Image quality and resolution were assessed in the middle of the field of view and at the edges of a 5.54 mm × 5.54 mm image plane. Resolutions were found to be consistently 0.78 µm in all measured locations, confirming no geometrical distortions and the high quality of the optics.

In a second step, a camera with a larger pixel size (9 µm; hr11002MTLGEC camera, SVS-Vistek GmbH, Seefeld, Germany) and field of view has been used. The resolution was again assessed in the middle and at the corners of the available image area. In the centre, the smallest resolved line was 2.46 µm, at the corners 3.1 µm. As this system was nearly able to resolve the 2.25 µm effective pixel size of the SVS camera, it should be able to resolve the larger 2.75 µm effective pixel size of the GigaFRoST camera (Mokso *et al.*, 2017 ▸) in use at the TOMCAT beamline.

### 2.4. Motorized aperture   

The novel macroscope features a motorized aperture placed at the exit pupil of the objective, to adjust the amount of light permitted through to the detector imaging objective and further to the detector camera. The aperture size can be tuned between 20 mm and 70 mm. While the nominal numerical aperture of the objective is 0.35, tuning the aperture size and reducing the amount of received light at the detector plane creates an effective numerical aperture that scales with the aperture size. The effective numerical aperture NA_eff_ can be calculated as NA_eff_ = *D*/(2*F*), where *D* is the aperture size and *F* is the objective focal length (100 mm in this case). The tuneable aperture size and effective numerical aperture have the benefit of adding more flexibility to the imaging system and so help to tune the combination of received light and depth of focus to push the spatial resolution of the imaging system to its maximum.

When the macroscope is operated with an aperture size of 70 mm, all received light will be passed to the detector objective and further to the detector camera. The macroscope has an effective numerical aperture of 0.35, which is also the maximum numerical aperture of the system. When the aperture size is reduced, the effective numerical aperture of the macroscope decreases. For the minimum aperture size of 20 mm, the effective numerical aperture is 0.1.

### 2.5. Flexible configuration   

The novel macroscope consists of three modules: the main objective module and two flexible modules at either end. The head module in the front of the macroscope can be rotated and oriented according to the needs of the experiment. The camera module at the back permits a highly flexible positioning of the detector, to ensure maximum stability of the setup. In the simplest configuration, the image plane is located after the tube lens at the rear of the macroscope. When the objective module is mounted vertically (Fig. 3 ▸), the detector follows then mostly on the top. The objective module can also be mounted horizontally (Fig. 3 ▸) for instance to be able to position tall samples close to the scintillator and prevent edge-enhancement artefacts in the acquired images. In this horizontal configuration, an additional rear bending mirror is usually added to redirect the light towards any side of the macroscope. (Heavy) detectors can then be secured in an easier and more stable manner.

## 3. Macroscope characterization   

### 3.1. Imaging setup   

The experiments were carried out at the TOMCAT beamline (Stampanoni *et al.*, 2006 ▸) at the Swiss Light Source (Paul Scherrer Institut, Switzerland). If not otherwise specified, the used monochromatic beam energy was 15 keV with approximately 2% bandwidth. During the experiments the novel macroscope was operated in the horizontal configuration and coupled with two different detector cameras and scintillators (more details in Sections 3.1.1 and 3.1.2) to investigate strengths and limitations of the novel imaging system for high-temporal- and high-spatial-resolution experiments. All samples were positioned close to the scintillator to minimize edge-enhancement.

The flexible endstation for tomographic microscopy at TOMCAT enables sample translation along three spatial directions with accuracy better than 1 µm. The 0.1 µm accuracy of the axis perpendicular to the beam direction and the sample centering stages assure artefact-free acquisition of reference images and high reproducibility of sample positioning, respectively. The custom-designed Aerotech air-bearing-based rotation axis system can rotate up to 10 Hz and has a run-out error of less than 1 µm at 100 mm from the rotation surface. Thanks to the accurate sample positioning and rotation capabilities, the system offers high reproducibility and is flexible and tuneable for a variety of sample setups. Moreover, the selection of available microscopes, detector cameras and scintillators enables investigation of a variety of samples covering a few orders of a magnitude in spatial and temporal resolutions. The generous space available around the sample stage allows *in situ* experiments with even more complicated setups exploiting additional hardware such as chambers and compression devices.

#### 3.1.1. Detectors   

Experiments were completed using two different cameras. The pco.Edge5.5 system (PCO AG, Kelheim, Germany) is built on sCMOS technology and it features a sensor with 2560 × 2160 pixels and a pixel size of 6.5 µm. With its moderate frame rate capability (100 frames s^−1^ at full frame), this detector is mainly devoted to research experiments where the focus lies primarily on spatial rather than temporal resolution.

The in-house-developed GigaFRoST camera (Mokso *et al.*, 2017 ▸) combines the CMOS chip of the pco.Dimax (PCO AG, Kelheim, Germany) with a novel readout system providing continuous data-streaming rates up to nearly 8 GB s^−1^. The sensor has 2016 × 2016 pixels with a pixel size of 11 µm. With its high frame rate (1255 frames s^−1^ at full frame) and its unique streaming capabilities, this detector is mainly used for time-resolved experiments.

The novel macroscope has a set magnification of 4×. Together with the pco.Edge5.5 detector camera the field of view covered by the macroscope is 4.2 mm × 3.5 mm and the pixel size 1.6 µm. Coupled with the GigaFRoST camera, the macroscope achieves a field of view of 5.5 mm × 5.5 mm and a pixel size of 2.75 µm.

#### 3.1.2. Scintillators   

Two different scintillators were coupled to the optics, both produced by Crytur (Turnov, Czech Republic). A thin 20 µm LuAG:Ce scintillator is mostly used for high-resolution imaging, while a thicker 100 µm LuAG:Ce scintillator is typically chosen for ultra-fast experiments with sub-second scan times.

For each imaging system configuration, the exposure time was tuned to achieve the same amount of counts on the flat-field images.

### 3.2. Spatial resolution   

To characterize the performance of the novel macroscope in terms of spatial resolution, we considered imaging setups combining both 20 µm and 100 µm LuAG:Ce scintillator screens with both available cameras. Furthermore, for each setup measurements with three different aperture sizes were performed. The spatial resolution of each system was estimated through the modulation transfer function and verified with a Siemens star.

#### 3.2.1. Modulation transfer function   

The modulation transfer function (MTF) (Boreman, 2001 ▸) is often used to measure the spatial resolution of an imaging system as it offers information about the magnitude response of the imaging system to sinusoids over a range of spatial frequencies. To quantify and compare the spatial resolution among different imaging systems, the frequency corresponding to the MTF at 10% was chosen as a reference value.

The MTF of the imaging system was estimated following the slanted edge technique (*e.g.* Fujita *et al.*, 1992 ▸; Stampanoni *et al.*, 2002 ▸; Palma-Alejandro *et al.*, 2013 ▸) where an edge is placed at a slight angle with respect to the horizontal detector plane and a radiograph is acquired. The acquired image of the edge is divided into blocks so that each block has one pixel displacement in the vertical direction while several pixels are included in the horizontal direction to achieve the displacement. From the block each horizontal row can then be used to create an oversampled edge spread function (ESF), where each image pixel is now represented by several values in the ESF. The line spread function (LSF) can by estimated by differentiation of the ESF. Moreover, the magnitude of the Fourier transform of the LSF results in the one-dimensional MTF.

The MTF was estimated by acquiring images of a copper slit (Plano, G220-7). The round sample had a radius of 2 mm, was approximately 10–12 µm thick and had a 1 mm-wide rectangular slit. The slit was positioned with a slight tilt of 1° with respect to the horizontal pixel row. The final MTF assessed resolution is the mean resolution estimate over ten images.

An in-house-manufactured Siemens star was used to verify the MTF assessed resolution. The golden Siemens star had a diameter of about 978 µm and a structure height of 1.5 µm. The linewidth of the spokes decreases gradually from 12 µm down to 150 nm, documented by the decreasing numbers in the Siemens star. Each number represents the line width (in µm) at the corresponding perimeter.

#### 3.2.2. Results   

**Figure 4:**
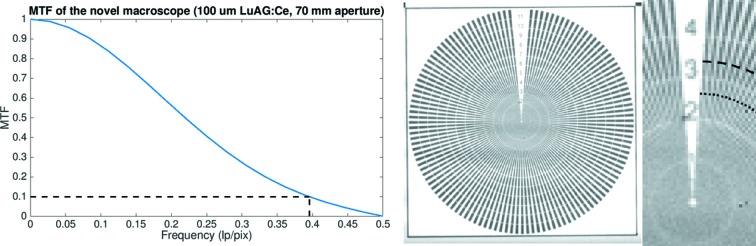
MTF curve (left) and Siemens star image (right) obtained for the novel macroscope coupled to the GigaFRoST, with a 100 µm LuAG:Ce scintillator and 70 mm aperture size. The dashed arc in the zoomed Siemens star image corresponds to the MTF estimated resolution. The theoretical maximum resolution (2 pixels) is marked with a dotted arc.

The frequency at the MTF 10% contrast was considered as the reference value to compare the spatial resolution between the different imaging setups. In Table 1 ▸, we report the estimated resolution for each combination of scintillators and aperture sizes when the macroscope was coupled to the GigaFRoST camera. In Fig. 4 ▸ we present, as an example, the MTF curve obtained for the highest resolution setup together with a Siemens star image to illustrate the correspondence between the MTF estimated resolution and the line pair visibility of the Siemens star. The correspondence between the estimated resolution and the line width in the Siemens star was calculated by multiplying the estimated MTF resolution with the pixel size, and by dividing the result by two to obtain the width of a single line, marked in the Siemens star by a dashed arc. The theoretical maximum resolution (2 pixels) is marked with a dotted arc.

As expected, we observed the maximum light on the camera sensor when using the 100 µm scintillator and an aperture size of 70 mm (Table 1 ▸). The resolution remained very stable when varying the aperture size: closing the aperture from 70 mm to 30 mm did not improve the spatial resolution, while the available light decreased by a factor of 5.5, as expected by the corresponding reduction in aperture area. Reducing the thickness of the scintillator did not increase the spatial resolution either. These observations indicate that for the configuration of the macroscope with the GigaFRoST camera the resolution is actually limited by the 11 µm pitch size of the imaging chip rather than the macroscope aperture size or scintillator thickness. The Siemens star images support the MTF estimated resolution, which turns out to represent a conservative value. We consider the 100 µm scintillator and a maximum aperture of 70 mm to be the most optimal imaging settings for the novel macroscope when coupled with the GigaFRoST camera, leading to a novel imaging system especially suited for the needs of time-resolved tomographic experiments requiring few micrometres spatial resolution.

**Figure 5:**
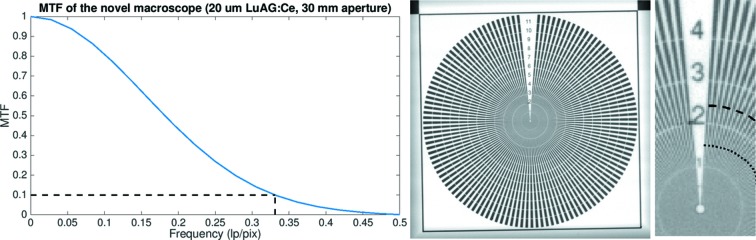
MTF curve (left) and Siemens star image (right) obtained for the novel macroscope coupled to the pco.Edge5.5, with a 20 µm LuAG:Ce scintillator and 30 mm aperture size. The dashed arc in the zoomed Siemens star image corresponds to the MTF estimated resolution. The theoretical maximum resolution (2 pixels) is marked with a dotted arc.

The same experiment was repeated for the macroscope coupled to the pco.Edge5.5 camera. This second setup featured a pixel size of 1.6 µm. Table 2 ▸ presents the resulting resolution values at 10% MTF for each combination of numerical apertures and scintillators. Fig. 5 ▸ shows the MTF curve for the best imaging setup together with the corresponding Siemens star images.

Thanks to the small pixel size offered by the pco.Edge, this imaging system benefits from a thinner scintillator and shows a resolution improvement when the 20 µm screen is used as opposed to the 100 µm. The highest resolution for both scintillator thicknesses is achieved when the aperture was closed to 30 mm. For high-spatial-resolution studies, we thus recommend the novel macroscope with a 30 mm aperture size and a 20 µm LuAG:Ce scintillator to achieve the highest spatial resolution. Increasing the aperture size to 70 mm can offer an efficiency increase by nearly a factor of 6, while the spatial resolution is compromised only by a factor of 1.1.

### 3.3. Distortions   

We used a copper mesh (Plano, G210) with 75 squared holes (size 320 µm) with a thickness of 12–15 µm to analyse possible distortions in each imaging setup. The mesh was mounted on the sample stage and aligned perpendicularly to the beam propagation. The mesh was then moved across the whole field of view and the acquired images of the mesh grid were compared with an artificial grid at each mesh position.

We did not observe any distortions for any considered imaging system configuration.

### 3.4. Efficiency difference between horizontal and vertical configurations   

To identify possible efficiency differences between the horizontal and vertical setup, a set of flat-field images was collected in both configurations. For this comparison, the macroscope was coupled with the GigaFRoST camera and both available scintillators were used. In addition to monochromatic radiation, the efficiency was also assessed using polychromatic radiation together with the 100 µm-thick scintillator. All experiments were performed with a fully open aperture at 70 mm.

As the results in Table 3 ▸ illustrate, there was only a slight efficiency loss of 2–4% when operating the macroscope in the horizontal setup. This loss is in agreement with the characteristics of the additional bending mirror, needed in horizontal configuration to redirect the light into the camera.

## 4. Comparison with other optical microscopes   

### 4.1. Optical descriptions   

The novel macroscope was compared with two optical systems available at TOMCAT, one dedicated to time-resolved experiments and one for high-quality tomographic scans.

A 2–4× white-beam compatible microscope (Elya Solutions, Prague, Czech Republic) coupled with the GigaFRoST camera has been the standard imaging setup at TOMCAT for fast, time-resolved tomographic experiments. This microscope features a commercial high-end photographic objective (Canon EF 85 mm f/1.2L II USM Lens) used in a reverse configuration. A continuously adjustable magnification from 2.24 to 3.78× is achieved by simultaneously modifying the distance between the objective and the scintillator on one side and the objective and the detector plane on the other side. The objective aperture can only be controlled electronically and has been set to 1.4 during the microscope assembly leading to an aperture size of 60 mm. This microscope can be used both with monochromatic and polychromatic radiation.

This setup was compared with the novel macroscope coupled to the GigaFRoST camera. In both cases a LuAG:Ce 100 µm scintillator was used, as is routinely done in time-resolved experiments. As the 2–4× microscope tuneable magnification can only be set to a maximum of 3.78×, these two imaging systems had a slightly different pixel size: 2.9 µm for the 2–4× optics and 2.75 µm for the novel macroscope.

The existing high-resolution standard setup at TOMCAT consists of a monochromatic beam dedicated microscope (Optique Peter, Lentilly, France) with interchangeable visible-light microscope commercial objectives (Olympus). The system can cover a wide magnification range from 1.25 to 40×. In this study we selected the 4× objective (with a numerical aperture of 0.16) coupled with the pco.Edge5.5 camera.

This setup was compared with the novel macroscope coupled to the pco.Edge camera. Both systems had the same pixel size of 1.6 µm. The used scintillator was a 20 µm-thick LuAG:Ce screen, the usual choice at TOMCAT when the spatial resolution and the image quality are more relevant than the temporal resolution.

### 4.2. Comparison results   

The different systems were compared in terms of distortions, spatial resolution, signal-to-noise and contrast-to-noise ratios.

#### 4.2.1. Resolution   

**Figure 6:**
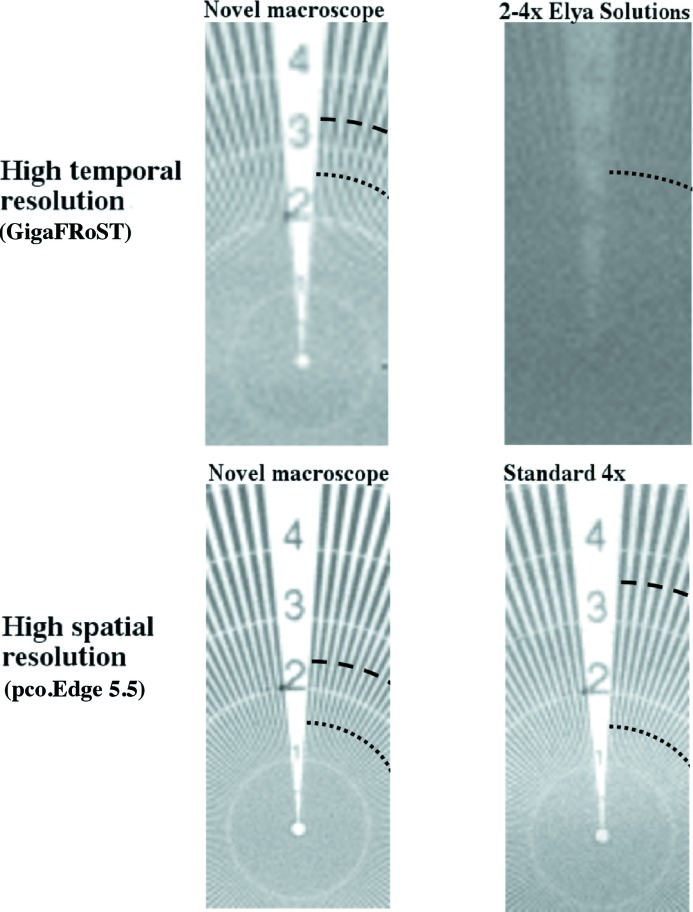
Flat-field-corrected Siemens star images for the different analysed imaging setups. The dashed arc corresponds to the MTF estimated resolution. The theoretical maximum resolution (2 pixels) is marked with a dotted arc. For the 2–4× Elya Solutions case, the MTF estimated resolution is out of the zoomed Siemens star resolution scale.

For the assessment of the spatial resolution for the two additional optical systems available at TOMCAT, the same procedure as detailed above has been followed. Siemens star images for all imaging setups under consideration are presented in Fig. 6 ▸ for illustration of the obtained image quality. The resulting 10% MTF values are presented in Table 4 ▸.

For the fast imaging setup exploiting the GigaFRoST camera, the novel macroscope provides an increase in resolution of nearly a factor of 6, confirming the extremely high quality of this new optical system. Moreover, the high efficiency of the novel macroscope leads to a reduction of the exposure time by more than a factor of 4 for the same amount of light on the sample. The dramatic reduction of resolution observed for the Elya Solutions microscope arises from the blurring-halo artefact introduced by the microscope optics and illustrated in Fig. 6 ▸. Due to the strongly blurred edge of the slit used for the resolution estimation and the visible halo around the object, the estimated edge spread function is broader, thus resulting in a very low resolution value.

For the high-resolution imaging setup, the novel macroscope increases the resolution by a factor of 1.5. When the novel macroscope is operated with an aperture size of 30 mm, its high efficiency leads to a reduction of the exposure time compared with the originally available system by a factor of 1.5. If we accept a slight (1.1×) compromise in resolution for the novel macroscope setup by increasing the aperture size from 30 mm to 70 mm, the exposure time can be reduced by nearly a factor of 8.5 in comparison with the standard microscope setup.

#### 4.2.2. Signal-to-noise and contrast-to-noise ratios   

The signal-to-noise ratio (SNR) and contrast-to-noise ratio (CNR) can be used to estimate the noise level of the image and contrast between different materials, respectively (Smith, 2003 ▸). Following the definition by Smith (2003 ▸), we define the SNR as the ratio between the mean and the standard deviation of the intensity in a selected region within an image. The CNR is defined as the ratio between the absolute difference of mean values and the sum of standard deviations of the intensities of two different regions within the image. Higher SNR and CNR values indicate lower noise level and better contrast between two different materials, respectively.

*Image quality in projection images.* The quality of the acquired and flat-field-corrected projection images was assessed using the slit described above. The SNR was measured in an area within the copper slit, the CNR between areas in the copper slit and air. In Table 5 ▸ we present the results of the SNR and CNR comparison for the high-temporal-resolution setup, where the GigaFRoST camera and a 100 µm-thick LuAG:Ce scintillator were coupled to the novel macroscope and to the 2–4× Elya Solutions microscope. The exposure time was tuned to have the same mean intensity value in flat-field images (approximately 2980) for both setups.

The results presented in Table 5 ▸ indicate that combining the GigaFRoST camera with the novel macroscope results in a higher SNR, by nearly a factor of 1.8, than with the 2–4× Elya Solutions microscope. Also the CNR was increased by a factor of 2.4 and exposure time reduced by nearly a factor of 5 when the novel macroscope was used.

The same protocol was used also to assess the projection quality of the high-spatial-resolution setups. The novel macroscope and the standard microscope with a 4× Olympus objective were both coupled to the pco.Edge5.5 camera with a 20 µm-thick LuAG:Ce scintillator. Exposure times were tuned to have the same average number of counts in flat-field images (approximately 46400) for all setups.

The results presented in Table 6 ▸ show that coupling the pco.Edge camera with the novel macroscope increases the SNR for the 30 mm aperture size by a factor of 1.6 and by a factor of 1.7 for the 70 mm aperture size compared with the standard microscope. The CNR increases for the 30 mm aperture by nearly a factor of 1.5 and for the 70 mm aperture by a factor of 1.4. The difference in both SNR and CNR between the two aperture sizes is minor.

**Figure 7:**
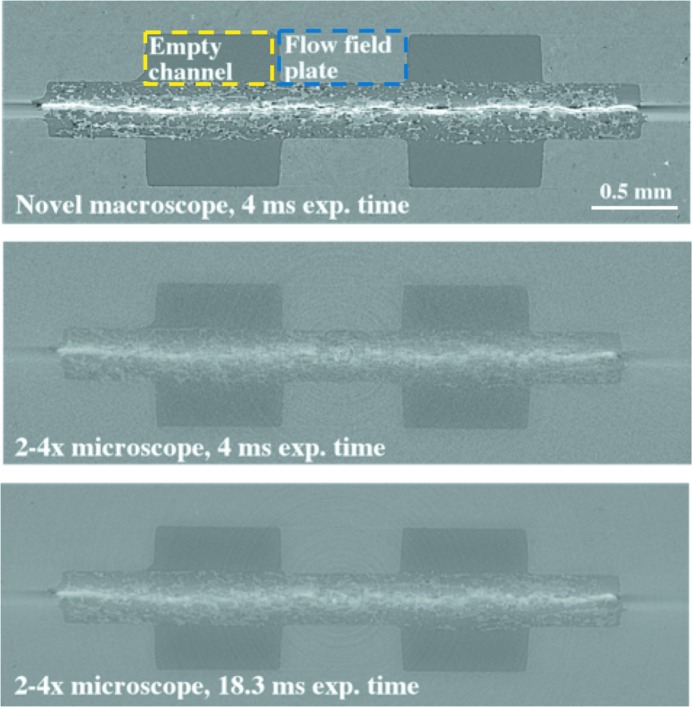
Tomographic slices of the fuel cell sample. Top: reconstruction from novel macroscope images and 4 ms exposure time. Middle: reconstruction from Elya Solutions microscope images and 4 ms exposure time. Bottom: reconstruction from Elya Solutions microscope images and 18.3 ms exposure time. All reconstructions were created from 1000 projection images. Grey levels are set equally for all images.

*Image quality in 3D.* The presented optical systems are mainly used for tomographic microscopy experiments where the novel macroscope will be critical for pushing time-resolved studies to the 10–20 Hz regime. We compared the reconstructed image quality for the setups for time-resolved investigations using a fuel cell sample, which is approximately 5 mm in diameter and perfectly fits the available field of view. The image quality of the tomographic volumes was assessed in terms of SNR and CNR, and an edge profile running across the graphite flow field plate and an empty channel was used to visualize the sharpness. The SNR was measured from an area in the empty channel close to the centre of the sample (yellow rectangle) and the CNR between areas in the empty channel and the flow field plate (blue rectangle) (Fig. 7 ▸).

We compared the noise and contrast level differences between the two fast imaging setups, the novel macroscope and the Elya Solutions microscope both with a 100 µm-thick LuAG:Ce scintillator and coupled to the GigaFRoST. All imaging experiments with the fuel cell were performed using monochromatic beam energy of 13.5 keV.

In a first step, a high-quality scan was assessed, where 1000 projection images were acquired with 4 ms exposure each. For the Elya Solutions microscope a second high-quality scan was also acquired. In this second case, the exposure time per projection was increased from 4 to 18.5 ms to achieve the same mean intensity value in flat-field images as for the novel optics.

**Figure 8:**
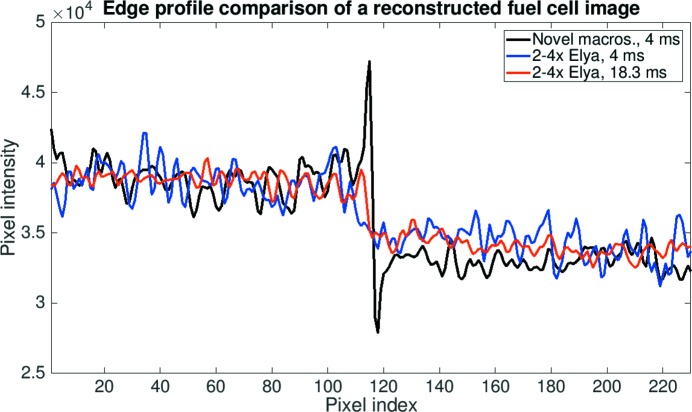
Edge profile comparison of single reconstructed fuel cell slices. The edge profile between the flow field plate and the empty channel is compared between the novel macroscope and the 2–4× microscope both for an exposure time of 4 ms as well as for an exposure time of 18.3 ms for the 2–4× microscope to have the same amount of light in the flat-field images as the more efficient novel macroscope.

The results presented in Table 7 ▸ indicate that the 4 ms exposed tomographic volumes acquired with the novel macroscope have a SNR nearly a factor of 2 higher than those obtained with the Elya Solutions microscope. By increasing the exposure time for the Elya Solutions optics by nearly a factor of 5 to equal the photon counts on chip to the new system, the SNR and CNR values reach slightly higher values than those for the novel macroscope. By comparing the reconstructed cross-section images in Fig. 7 ▸ and edge profile plots between the flow field and an empty channel in Fig. 8 ▸, it is however evident that the reconstructed image quality is actually not superior in comparison with the novel macroscope, but the halo-blurring artefact acts as a smoothing filter which artificially leads to higher SNR and CNR measures. In Fig. 8 ▸, the difference in edge sharpness in the profiles for the two optical systems is obvious. The profile for the novel macroscope displays a sharp knife-like edge while the edge profiles for the Elya Solutions microscope are significantly more blurred, with the edge for the 4 ms exposure time case being the most blurred. For the 18.3 ms exposure time case, the smoothing-effect of the halo-blurring artefact is clearly visible along the entire profile. The line is overall smoother than the profile for the novel macroscope, which instead resolves the microstructures of the graphite flow field plate. The superior resolution of the new macroscope leads to an artificial decrease in CNR, if the imaged material is not completely homogeneous at the micrometre scale, as is often true. The smoothing-effect of the halo-blurring artefact cannot counteract the high noise level for the 4 ms case, due to the reduced number of photons reaching the detector. The profile for the 4 ms case shows an increased roughness, which cannot be attributed to the microstructure but to the high noise level. The high peak at the edge in the novel macroscope profile is due to edge-enhancement. These fringes are not resolved by the Elya Solutions microscope.

In a second step, the performance of the optical systems in the sub-second scan regime, most relevant for dynamic studies, has been assessed. We compared 0.4 s, 0.2 s and 0.1 s scan time reconstructions, each having 400 projections and exposure time varying between 1 ms, 0.5 ms and 0.25 ms, respectively.

**Figure 9:**
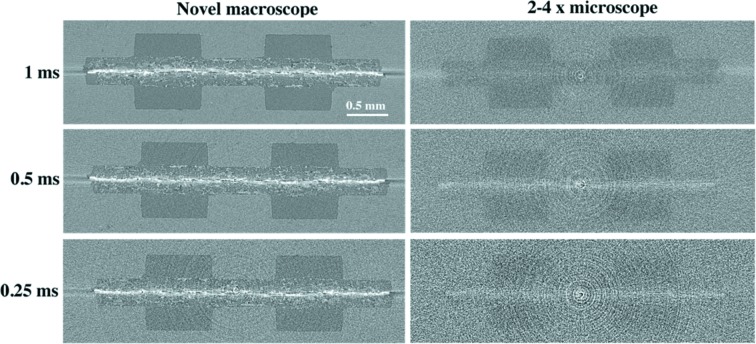
Tomographic slices of the fuel cell sample. Left: reconstructions from novel macroscope images. Right: reconstructions from Elya Solutions microscope images. For both imaging setups the projection exposure time was from top to bottom 1 ms, 0.5 ms and 0.25 ms. All reconstructions were created from evenly spaced 400 projections images.

The novel optical system outperforms the Elya Solutions microscope for all sub-second scan settings, with the image quality difference increasing with decreasing scan time (Table 8 ▸). The tomographic reconstruction for the 0.1 s scan acquired with the novel macroscope shows a high SNR of 6.0, while for the same scan settings the SNR obtained with the Elya Solutions microscope is 2.2. The CNR of 0.4 obtained with the novel macroscope is also 4 times higher than for the older microscope. Considering the 4-times-higher efficiency of the novel macroscope, the 0.1 s scan obtained with the novel macroscope is comparable with the 0.4 s scan obtained with the old system in terms of light collected on chip. Under these scan settings, the 2–4× microscope and the novel macroscope reach nearly equal SNR while the novel macroscope delivers slightly higher CNR by a factor of 1.3. However, as evident from Fig. 9 ▸, the small fibres in the gas diffusion layers are not resolved with the old system due to noise and smoothing effects while they are still distinguishable with the novel macroscope. The high noise level and smoothing artefact of the sub-second tomographic volumes acquired with the Elya Solutions optics cause severe challenges for image processing and further image analysis. The novel macroscope and its robust image quality will be crucial for expanding the imaging possibilities in the sub-second regime at the TOMCAT beamline.

## 5. Discussion and conclusion   

We have introduced an improved imaging system based on a novel, high-resolution macroscope. When it is coupled with the GigaFRoST camera, the setup is optimal for high-resolution (10–20 Hz) time-resolved studies with a pixel size of 2.75 µm; when it is coupled with the pco.Edge5.5, the excellent image quality of the system and its relatively small pixel size (1.6 µm) provide excellent conditions for fast high-resolution scans. This feature is particularly important for the investigation of centimetre-size samples at high resolution, where hundreds of millimetre-cubed high-resolution tomographic sub-volumes need to be acquired to cover the entire specimen (*e.g.* Gonzalez-Tendero *et al.*, 2017 ▸).

For fast sub-second imaging studies, the novel macroscope should be coupled to the GigaFRoST and a 100 µm-thick LuAG:Ce scintillator and the aperture should be completely open (70 mm). We observed that decreasing the aperture size and scintillator thickness did not result in increased spatial resolution, as the resolution in this case is limited by the GigaFRoST chip pitch size. For high-spatial-resolution studies, the novel macroscope with a 30 mm aperture size should be coupled to the pco.Edge5.5 and a 20 µm-thick LuAG:Ce scintillator. Increasing the aperture size to 70 mm can offer an efficiency increase of nearly a factor of 6 with just a minimal spatial resolution reduction (factor of 1.1).

Thanks to the high efficiency of the novel macroscope, the exposure times for high-temporal-resolution experiments can be decreased by a factor of 4 and for high-spatial-resolution experiments by nearly a factor of 8.5 compared with the setups previously used at TOMCAT. The new optics is not only significantly more efficient but at the same time it also features a superior image quality compared with the previous systems in-house. The spatial resolution reached in time-resolved studies is up to 6 times higher than when the Elya Solutions microscope, suffering from a blurring-halo artefact, is used. The spatial resolution is also superior, by a factor of 1.5, for high-resolution investigations. The high efficiency, spatial resolution and image quality provided by the novel macroscope will enormously expand the systems that can be investigated with high-temporal-resolution and will make 10–20 Hz studies routinely possible.

The achieved high image quality and high efficiency do not only lead to an increased temporal resolution for ultra-fast tomographic experiments but also promote a significant reduction in radiation dose, an aspect beneficial also (and especially) at upcoming brighter sources. It is becoming increasingly clear that also non-biological systems (such as fuel cells, batteries and water contact angles in porous media) are adversely affected by X-ray irradiation. A high NA system such as the one presented here, where an increased number of photons which have interacted with the sample are actually collected by the optics, is ideal to optimize the balance between time resolution and dose reduction depending on the performed experiment.

For high-energy imaging experiments (currently not available at TOMCAT), typically thicker scintillators are used to ensure sufficient light at the camera chip. Increasing the scintillator thickness will deteriorate the spatial resolution of any imaging system, as demonstrated in Table 2 ▸, if the resolution is not limited by the sensor pitch size (Table 1 ▸). However, the high efficiency of the novel system will ensure a shorter exposure time, helping to reduce the radiation on the sample and the needed scan time. Alternatively, the high light collection efficiency should enable the reduction of the screen thickness, while maintaining comparable chip illumination statistics as standard systems, leading to an increase in the final spatial resolution.

The novel macroscope is not limited to beamline applications, but could also be coupled to a table-top tomography system when a small field of view is used. While the macroscope presented here has been designed especially for the TOMCAT beamline (including polychromatic applications), the high image quality and high efficiency it offers could unlock new opportunities for table-top systems as well. If the numerical aperture of such existing systems is known, a theoretical estimation of the efficiency increase could be readily calculated.

Having seen the exceptional performance of this novel, high-resolution optical component as well as the new opportunities being opened up in the tomographic investigation of time-resolved and radiation-sensitive systems, it is not intended for this macroscope to be and remain a one-of-its-kind. The highly modular design and the tandem solution have in fact already been conceived with potential future applications and evolutions in mind. Building upon this first experience, developments towards commercially available new high NA systems also for different higher and lower magnifications are envisaged. Developments towards a near ultraviolet (NUV) version of the macroscope are also planned. Recently developed scintillators emitting in the NUV spectrum will at the same time push the resolution limit of the imaging system and, thanks to their short decay time and limited afterglow effects, increase the quality (and consequently spatial resolution) of tomographic volumes acquired with a high time resolution.

## 6. Conflict of interest statement   

XR is the president of the Optique Peter company, which has developed, manufactured and delivered the presented novel macroscope. The other authors declare no conflict of interest.

## Figures and Tables

**Table 1:** Comparison of the estimated resolution of the novel macroscope coupled to the GigaFRoST camera with different scintillators and aperture sizes The exposure time was tuned to have the same counts (approximately 2980) for the flat-field images for all configurations. With the 20 µm scintillator, the minimum numerical aperture applicable was 40 mm, because for a smaller aperture the maximum exposure time of the GigaFRoST (40 ms) did not allow to reach the same amount of counts on flat-field images as for the other cases.

Scintillator	Aperture size	Exposure time	Resolution (MTF 10%)	Standard deviation
LuAG:Ce 100 µm	70 mm	4.2 ms	2.5 pixels	0.2
	30 mm	23.1 ms	2.6 pixels	0.1
LuAG:Ce 20 µm	70 mm	8.5 ms	2.6 pixels	0.1
	40 mm	30 ms	2.6 pixels	0.2

**Table 2:** Comparison of the estimated resolution of the novel macroscope coupled with the pco.Edge5.5 camera with different scintillators and aperture sizes The exposure time was tuned to have the same counts (approximately 46400) for the flat-field images for all configurations.

Scintillator	Aperture size	Exposure time	Resolution (MTF 10%)	Standard deviation
LuAG:Ce 100 µm	70 mm	9 ms	4.7 pixels	0.2
	30 mm	50 ms	3.4 pixels	0.4
LuAG:Ce 20 µm	70 mm	22 ms	3.4 pixels	0.1
	30 mm	123 ms	3.0 pixels	0.1

**Table 3:** Efficiency difference between horizontal and vertical configurations Exposure times were tuned to obtain the same mean intensity value in flat-field images for all configurations. Under polychromatic radiation the maximum exposure time before saturation was 1.1 ms and therefore applied for the vertical setup. Moving to the horizontal setup, the exposure time increased to 1.14 ms before saturation.

Camera	Radiation	Scintillator	Horizontal exposure time	Vertical exposure time	Mean value
GigaFRoST	Monochromatic (15 keV)	20 µm	8.0 ms	7.8 ms	2955
		100 µm	3.87 ms	3.7 ms	2955
	Polychromatic	100 µm	1.14 ms	1.1 ms	2777

**Table 4:** Resolution and efficiency comparison between the existing and novel optical systems for high temporal and high spatial resolution imaging setups

	Camera	Scintillator	Microscope	Pixel size	Aperture	Exposure time	Resolution (MTF 10%)	Standard deviation
High temporal resolution	GigaFRoST	LuAG:Ce 100 µm	2–4× Elya	2.9 µm	60 mm	19.5 ms	14.9 pixels	0.2
			Novel macroscope	2.75 µm	70 mm	4.2 ms	2.5 pixels	0.2

High spatial resolution	pco.Edge5.5	LuAG:Ce 20 µm	Standard 4×	1.6 µm	Maximum	185 ms	4.5 pixels	0.1
			Novel macroscope		30 mm	123 ms	3.0 pixels	0.1
					70 mm	22 ms	3.4 pixels	0.1

**Table 5:** SNR and CNR comparison for flat-field-corrected projection images for the high-temporal-resolution setups exploiting the GigaFRoST camera and a 100 µm thick LuAG:Ce scintillator

Microscope	Aperture	Exposure time	SNR	CNR
2–4× Elya	60 mm	19.5 ms	35.4	12.2
Novel macroscope	70 mm	4.2 ms	63.9	29.5

**Table 6:** SNR and CNR comparison for flat-field-corrected projection images for the high-spatial-resolution setups exploiting the pco.Edge5.5 camera and 20 µm-thick LuAG:Ce scintillator

Microscope	Aperture	Exposure time	SNR	CNR
Standard 4×	Maximum	185 ms	42.8	20.3
Novel macroscope	30 mm	123 ms	70.4	30.2
	70 mm	22 ms	71.6	28.7

**Table 7:** SNR and CNR comparison in tomographic volumes for the time-resolved tomography setup using the novel macroscope and the Elya Solutions microscope

Number of projections	Exposure time	Microscope	SNR	CNR
1000	4 ms	Novel macroscope	44.6	2.4
		2–4× Elya	24.2	1.5
	18.3 ms	2–4× Elya	49.1	3.1

**Table 8:** SNR and CNR comparison for tomographic volumes resulting from sub-second scans using the novel macroscope and the Elya Solutions microscope

Number of projections	Exposure time	Microscope	SNR	CNR
400	1 ms	Novel macroscope	14.0	1.0
		Elya 2–4×	6.2	0.3
	0.5 ms	Novel macroscope	9.9	0.7
		Elya 2–4×	3.5	0.2
	0.25 ms	Novel macroscope	6.0	0.4
		Elya 2–4×	2.2	0.1
